# High-fidelity multimode fibre-based endoscopy for deep brain in vivo imaging

**DOI:** 10.1038/s41377-018-0094-x

**Published:** 2018-11-21

**Authors:** Sergey Turtaev, Ivo T. Leite, Tristan Altwegg-Boussac, Janelle M. P. Pakan, Nathalie L. Rochefort, Tomáš Čižmár

**Affiliations:** 1 Leibniz Institute of Photonic Technology, Albert-Einstein-Straße 9, Jena, 07745 Germany; 2 School of Life Sciences, University of Dundee, Nethergate, Dundee, DD1 4HN UK; 3 School of Science and Engineering, University of Dundee, Nethergate, Dundee, DD1 4HN UK; 4 Centre for Discovery Brain Sciences, University of Edinburgh, Hugh Robson Building 15, George Square, Edinburgh, EH8 9XD UK; 5 Center for Behavioral Brain Sciences, Institute of Cognitive Neurology and Dementia Research, German Center for Neurodegenerative Diseases, Leipziger Straße 44, Haus 64, Magdeburg, 39120 Germany; 6 Simons Initiative for the Developing Brain, University of Edinburgh, Edinburgh, EH8 9XD UK; 7 Institute of Scientific Instruments of CAS, Kralovopolska 147, Brno, 612 64 Czech Republic

## Abstract

Progress in neuroscience relies on new techniques for investigating the complex dynamics of neuronal networks. An ongoing challenge is to achieve minimally invasive and high-resolution observations of neuronal activity in vivo inside deep brain areas. Recently introduced methods for holographic control of light propagation in complex media enable the use of a hair-thin multimode optical fibre as an ultranarrow imaging tool. Compared to endoscopes based on graded-index lenses or fibre bundles, this new approach offers a footprint reduction exceeding an order of magnitude, combined with a significant enhancement in resolution. We designed a compact and high-speed system for fluorescent imaging at the tip of a fibre, achieving a resolution of 1.18 ± 0.04 µm across a 50-µm field of view, yielding 7-kilopixel images at a rate of 3.5 frames/s. Furthermore, we demonstrate in vivo observations of cell bodies and processes of inhibitory neurons within deep layers of the visual cortex and hippocampus of anaesthetised mice. This study paves the way for modern microscopy to be applied deep inside tissues of living animal models while exerting a minimal impact on their structural and functional properties.

Optical systems have traditionally been understood as assemblies of components acting in a predefined and determinate manner. This notion is currently undergoing a transformation due to rapid advances in the technology and methods for spatial light modulation. Computer-controlled holographic modulators now facilitate the deployment of unusual and complex optical media with properties that bring unique advantages to biomedical applications^1^.

The use of multimode fibres (MMFs) as ultranarrow endoscopes is a promising example of this concept^2–10^, since it allows one to overcome the trade-off between the size of the optical element and the attainable resolution^11^. The nature of light transport through MMFs leads to the transformation (or scrambling) of incident wavefronts into seemingly random speckle patterns. Adaptive optics provides a means for overcoming this signal degradation^12–15^. A number of recently developed techniques in this domain have enabled the randomised output fields to be tailored into any desired distribution across the distal fibre facet or an arbitrarily remote plane^2–5^. Laser scanning microscopy utilised in this study relies on the formation of diffraction-limited foci behind the fibre, combined with image reconstruction from fluorescence signals that are collected and guided backwards^4,7^.

Digital micromirror devices (DMDs) have recently opened up a range of opportunities in this domain by increasing the achievable light modulation refresh rates by several orders of magnitude compared to well-established nematic liquid crystal-based devices^16–19^. As a result, the foci behind a MMF can now be scanned at several tens of kHz, thus acquiring images at speeds approaching video rates. Furthermore, it has also been shown that DMDs generate foci of higher quality compared to other modulators^20^. Building on these advances, the focus of researchers is currently shifting towards implementations in biomedically relevant settings, including in vivo applications^21^.

Here we devise a compact and high-speed imaging system capable of resolving micron-sized subcellular neural structures in an anaesthetised animal model through, to our knowledge, the most minimally invasive endoscopic probe reported thus far. Relying on high-performance holographic methods and a carefully optimised design, this represents the first instrument capable of time-lapse artefact-free imaging of both neuronal somata and dendritic processes with diffraction-limited resolution. As exemplified by optogenetics^22^, the extremely reduced footprint allows observations unprecedently deep within brain tissues, thus enabling a new level of in vivo investigation of neuronal connectivity in previously inaccessible brain structures.

The system consists of laser, calibration, beam-shaping and sample modules, as illustrated in Fig. 1a. A DMD in the core of the beam-shaping module is employed in the off-axis regime, and thus, despite its inherent binary-amplitude light modulation, can control the phase of the optical fields coupled into the fibre. As shown previously^3,4,20^, employment of the DMD and the calibration module allows characterising the light propagation throughout the entire optical path, from the DMD chip to a desired focal plane behind the distal fibre facet, which can be expressed as a transmission matrix (TM) of the system^14,15^. The TM allows the calculation of phase modulations, which, when applied to the DMD, result in the formation of diffraction-limited foci at any desired location in the focal plane. The sequential exposure of a fluorescent sample to such a series of foci, performed as the fluorescent signals propagating backwards through the fibre towards a bucket detector are collected, is equivalent to raster-scanning laser microscopy.

**Fig. 1:**
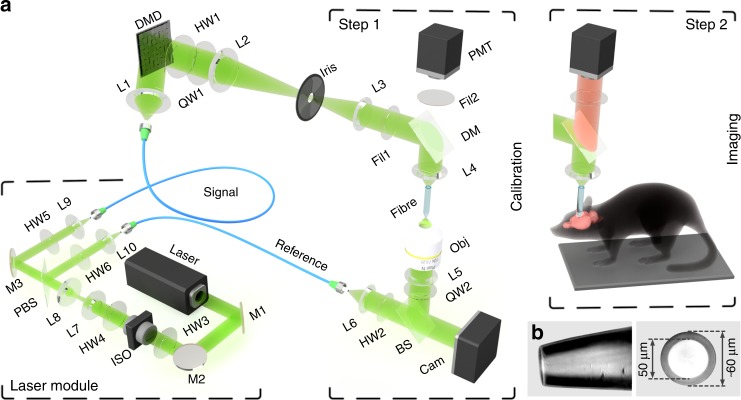
Multimode fibre-based imaging system. **a** Scheme of the imaging setup. The corresponding list of components is provided in Methods. A calibration (step 1) precedes imaging (step 2). During *in vivo* imaging, the mouse is head-fixed and placed under the fibre. The fibre is then lowered into the brain region of interest using a motorised stage. **b** Flat-top cone termination of the MMF probe with a core diameter of 50 µm and an external diameter of 60 µm

A standard commercially available MMF with a core of 50 µm in diameter and a NA of 0.22 was chosen as the endoscopic probe. To minimise the tissue damage caused by compression during the penetration process^23,24^, the fibre (2 cm in length) was postprocessed into a flat-cone termination by polishing the excess cladding from an external diameter of 125 µm to 60 µm, as depicted in Fig. 1b. In all experiments, we offset the focal plane 5–15 µm from the distal fibre facet to guarantee that the full NA of the fibre was reached uniformly across the field of view and to minimise sample-induced aberrations. Once the probe is placed and aligned with the system, it takes approximately 2 min to obtain the TM, compute ~7000 phase modulation patterns for all desired foci across a 50-µm-wide circular region of the focal plane, and upload the patterns to the memory of the DMD.

The fidelity of the generated foci determines the imaging quality. When controlling light propagation through a randomising medium, not all of the optical power leaving the medium can be directed to a diffraction-limited focus. The remaining power forms a background signal in the form of speckle, as shown in Fig. 2b. The ratio between the power in the focal spot and the total output power emitted from the fibre is commonly used as a figure of merit when assessing the performance of a given optimisation approach. Working in the off-axis regime and utilising circularly polarised light, this power ratio has been shown to reach 75%, which is very close to the fundamental limit for approaches based on phase-only modulation^25^. The enhancement factor, defined as the ratio of the peak intensity at the focal point to the average level of speckled background, is also commonly used to assess the quality of focussing; this factor exceeds 3100 in our experiments (see Methods for more details).

**Fig. 2:**
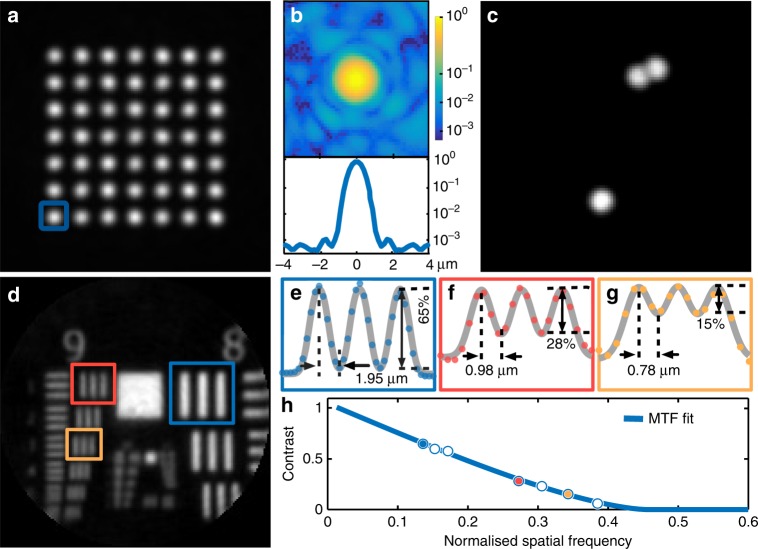
Evaluation of the imaging performance. **a** Combined foci (sum projection) at the distal MMF facet demonstrating the power uniformity of focusing at different locations. **b** Intensity distribution and azimuthally averaged profile of a chosen focal point (indicated by a blue square in panel a) in logarithmic scale. **c** Validation of fluorescent imaging using 4-µm fluorescent particles. **d**–**h** Assessment of spatial resolution using a negative USAF-1951 test target. Image of the test target (**d**) and contrast measurements of selected target elements with 265 line pairs per millimetre (lp/mm) (**e**), 512 lp/mm (**f**), and 645.1 lp/mm (**g**). **h** Imaging contrast as a function of the normalised spatial frequency for the target elements in (**d**), and the fit of the modulation transfer function (MTF) corresponding to an aberration-free imaging system with a circular aperture. The fit allows an estimation of the effective numerical aperture (NA) as 0.225 ± 0.008 and the corresponding resolution limit (Abbe criterion) as 1.18 ± 0.04 µm

Once the calibration procedure is finished, the calibration module is removed and exchanged with the sample module. A fast bucket intensity detector triggered by DMD reference TTL pulses (synchronised with switching between individual DMD modulations) allows the system to operate at a refresh rate of 23 kHz, which results in an imaging rate of ~3.5 frames/s. The calibration remains valid provided that the optical system does not suffer from mechanical or environmental factors, which may lead to perturbations in the light transport through the MMF and degradation of the imaging quality. As recently demonstrated, temperature changes in short fibre segments lead to an axial shift of the focus away from the fibre facet. At the scale of fibre lengths used in this study, the temperature change from room temperature to that of the animal body leads to a negligible axial shift not exceeding the Rayleigh range of the focused beam. Bending of the fibre may be another source of imaging performance degradation; however, at the curvature levels expected in our study, these effects are also negligible. The most severe calibration degradation is therefore to be expected from drifts in the optical pathway, particularly the mutual alignment of the DMD and MMF input. The system has therefore been devised with a robust cage-based construction, resulting in stable operation over several hours without the need for recalibration. The optical path was designed to achieve the maximum resolving power of the MMF.

The resolution of the system was assessed via a negative USAF 1951 test target. As shown in Fig. 2d–h, line separations of 3.9 µm, 1.9 µm, and 1.6 µm were imaged with contrasts of 65%, 28%, and 15%, respectively, which is in good agreement with the resolution limit (Abbe criterion) dictated by the wavelength and NA of the fibre. To validate the operation in the fluorescent regime (Fig. 2c), we used 4.0-µm red fluorescent beads on a microscope coverslip.

To demonstrate in vivo imaging capability, we used transgenic mice with a subpopulation of inhibitory interneurons labelled with a red fluorescent marker (see Methods). The fibre probe was inserted into the primary visual cortex (V1) and deeper into the hippocampus of the mouse brain through a small craniotomy. The images presented in Fig. 3a, b were recorded at different depths within V1 (0.5–0.8 mm) and in the CA1 region and dentate gyrus (DG) of the hippocampus (approximately 1.5 and 2 mm). The fibre probe causes minimal damage in vivo, as demonstrated by a postmortem section of a perfused brain after fibre imaging (Fig. 4b), showing the impact of the insertion procedure with a fibre tract width not exceeding 50 µm. The resolution and contrast achieved in vivo allow for visual identification of both relatively large objects, such as cell somata (diameter of approximately 10–20 µm, Fig. 3a), and thin processes (usually 1–2 µm wide) with fine structures corresponding to synaptic boutons (Fig. 3b). In addition to imaging anatomical structures in deep brain regions, the system can also be used to image dynamic processes over time, as exemplified by the observation of a minuscule local haemorrhage from a burst blood vessel, showing individual red blood cells (see Fig. 3c). The high frame rate of the system allows live recording of the labelled structures during fibre penetration (see Supplementary Movie).

**Fig. 3:**
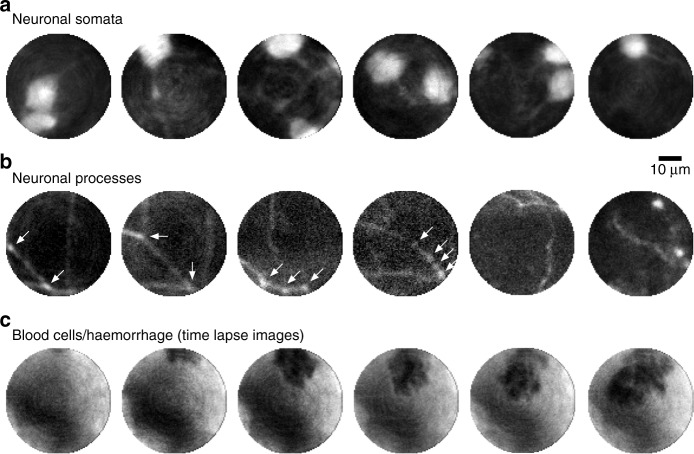
In vivo implementation of the MMF-based imaging system. Images of neuronal somata (**a**) and processes (**b**) of inhibitory neurons observed during a direct insertion of the MMF probe 2–3 mm into the mouse brain. The arrows indicate branching points and synaptic boutons. **c** Time-lapse images of a haemorrhage in the primary visual cortex: nonlabelled blood cells (dark) exiting a burst blood vessel are visible. The images were acquired at 3.5 frames/s. The frames displayed from left to right were acquired at 0 s, 0.57 s, 1.14 s, 1.71 s, 2.5 s and 4.6 s, respectively

**Fig. 4:**
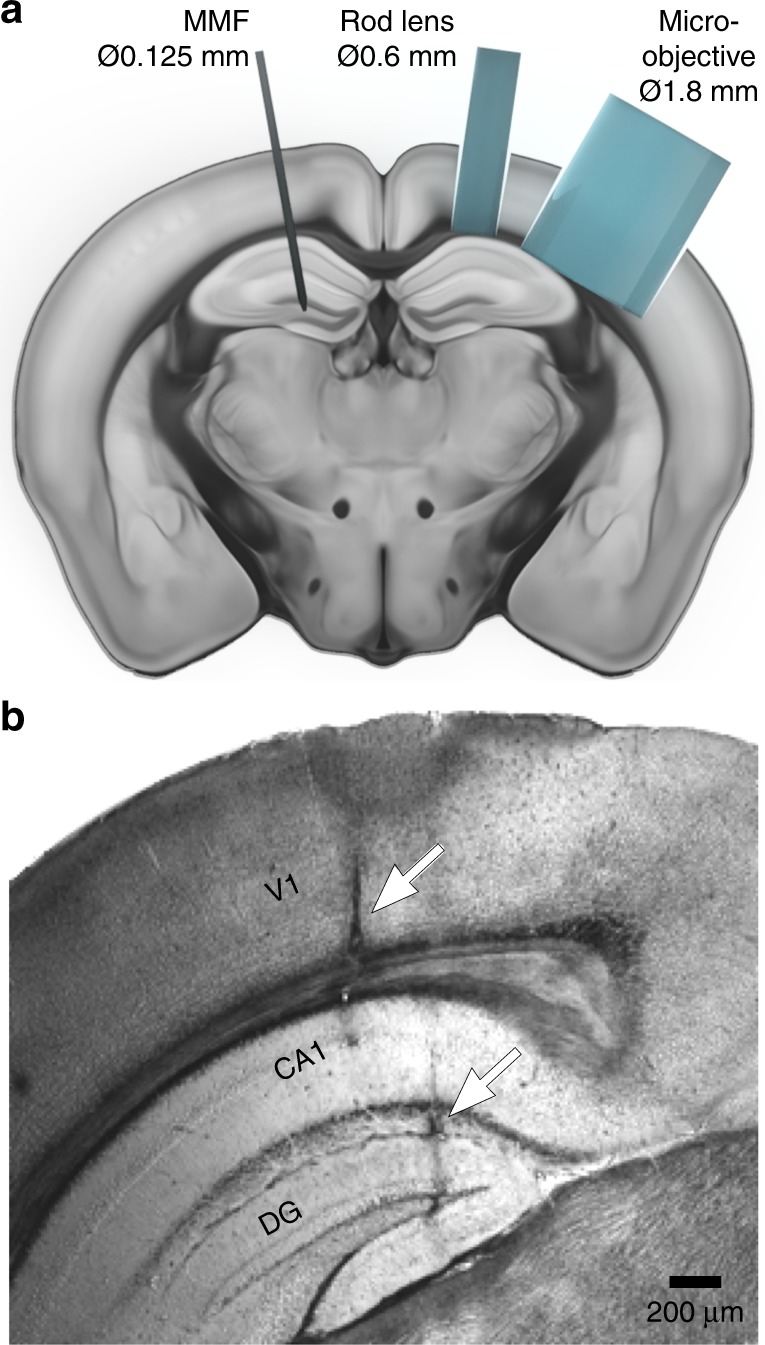
Assessment of invasiveness. **a** Scale-preserved comparison of common endoscopic probes and a MMF. **b** Postmortem coronal brain section showing two fibre tracts of the MMF probe in the visual cortex and hippocampus. The arrows indicate the insertion tract of the MMF probe. The width of each tract is less than 50 µm. Scale bar, 200 µm

In this work, we have designed a highly optimised optical pathway for fluorescence-based imaging of deep brain structures with micrometric spatial resolution while causing minimal damage to the tissue surrounding the fibre penetration area. The robust design allows continuous imaging for periods of several hours. Deploying the most efficient wavefront-shaping algorithms and, currently, the fastest possible hardware for light modulation, our system is capable of acquiring 7-kilopixel images with micron-level spatial resolution at imaging speeds of 3.5 frames/s. This provides adequate spatial and temporal resolution for fluorescent imaging of subcellular structures in living tissues.

We were able to perform fluorescence imaging in vivo deep within a mouse brain using a single MMF-based probe. In anaesthetised transgenic mice with tdTomato fluorescent protein expression in a sparse subpopulation of inhibitory interneurons, we obtained visually identifiable images of neuronal somata and processes in both the visual cortex and the hippocampus, >2 mm below the surface of the brain. While the imaging was restricted to the cortex and hippocampus in our experiments, with this technique, the entire dorsal-ventral extent of the mouse brain (4–6 mm) can be covered, allowing imaging of even the most ventrally located nuclei in the brain. The achieved resolution is limited only by the numerical aperture of the fibre, being comparable to a standard ×10 microscope objective. Imaging immediately after insertion of the endoscopic probe, which is only possible due to its minuscule footprint, eliminates the need for a long postoperative recovery period, as well as the necessity of surgical removal of overlying tissue and implantation of imaging elements, such as a graded-index lens^26,27^. The demonstrated capability to record dynamic processes with high resolution can be further extended to visualisation of structural changes (e.g., spine turnover) over extended periods of time.

Although the structures of interest in this report are clearly recognisable, the images exhibit a visible level of background. This background signal originates from sample excitation by out-of-focus light, and in the case of MMF-based imaging, the resulting glare features concentric wavelets due to the discrete structure of allowed propagating modes. Eliminating out-of-focus light by means of confocal rejection of incoherent fluorescent signals returning from the MMF is currently not possible without prohibitive power losses. Therefore, future work will focus on the development of computational postprocessing algorithms to further enhance the imaging quality.

This demonstration paves the way for in vivo implementation of numerous techniques of modern microscopy, including multiphoton^9^, super-resolution and light-sheet approaches^10^. Future advancements will strongly rely on the development of new fibre types directly optimised for the purposes of holographic endoscopy^11^.

## Methods

### Setup

As illustrated in Fig. 1a, the system is designed in a modular manner, consisting of laser, calibration, beam-shaping and sample modules. In the laser module, the beam is divided into a signal and a reference beam, which are coupled to single-mode polarisation-maintaining fibres. The calibration module is used to measure the transmission matrix of the multimode fibre, and is subsequently replaced by a sample module for the anaesthetised animal model.

#### Laser module

A single-frequency, diode-pumped solid-state laser source emitting at the wavelength of 532 nm provides a continuous-wave and linearly polarised beam. Achromatic doublets L7 and L8 form a telescope to demagnify the beam, and aspheric lenses L9 and L10 couple the signal and reference beams into the polarisation-maintaining fibres. Half-wave plate HW3, in combination with the input linear polariser of the Faraday optical isolator ISO, controls the combined power coupled to the signal and reference beams, whereas half-wave plate HW4, in combination with polarising beam splitter PBS, controls the power ratio between the signal and reference beams. Half-wave plates HW5 and HW6 are used to finely align the polarisation of the coupled beams with one of the birefringent axes of the polarisation-maintaining fibres (Fig. 1a).

#### Beam-shaping module

Achromatic doublet L1 collimates and expands the signal beam to overfill the DMD at an incidence angle of 24° with respect to the DMD chip. In this way, each micro-mirror in the ‘on’ state (+12°) reflects light in the direction of the optical system, whereas micro-mirrors in the ‘off’ state (−12°) redirect light towards a beam dump. Lenses L2, L3 and L4 relay the far-field of the first diffraction order of the holograms generated at the DMD to the proximal facet of the multimode fibre. An iris diaphragm is used in the Fourier plane of L2 to isolate the first diffraction order of the holograms, while blocking the remaining orders. The combination of lenses L2 and L3 is chosen to underfill the aperture of aspheric lens L4 in order to reduce its effective numerical aperture to 0.23, matching that of the multimode fibre. The multimode fibre used has a core diameter of 50 µm and a NA of 0.22 and sustains approximately 2100 propagation-invariant modes (i.e., 1050 for each orthogonal polarisation state) at the wavelength of 532 nm. Circular polarisation is well preserved after propagation through a straight fibre segment. The transmission matrix is measured between 3000 input modes and 7000 output modes (corresponding to the desired foci). Oversampling is essential to reach the highest fraction of optical power contained in a focused spot. Dichroic mirror DM, combined with excitation filter Fil1 and emission filter Fil2, separates and spectrally purifies the fluorescence signal. Photomultiplier tube PMT measures the overall intensity of the fluorescence signal. Half-wave plate HW1 and quarter-wave plate QW1 provide the two degrees of freedom necessary to reach the purest circular polarisation state of the spatially modulated signal at the input facet of the fibre.

#### Calibration module

A movable, compact calibration module is used to acquire the TM of the fibre prior to image acquisition. The TM is measured interferometrically using an external phase reference. Being uniformly distributed, the external reference prevents the formation of ‘blind spots’ originating from the speckled nature of internal references^3^. Microscope objective OBJ and tube lens L5 image the selected focal plane (spatially offset from the fibre facet) onto the CCD camera with a demagnification of 27.7 fold. The reference beam is collimated by aspheric lens L6 and combined with the signal beam using nonpolarising beamsplitter cube BS. Quarter-wave plate QW2 converts the polarisation state of the output speckle patterns to linear, and half-wave plate HW2 aligns the polarisation axis of the reference signal in order to maximise the signal-to-noise ratio of the interference patterns at the camera. The microscope objective is mounted on a single-axis translation stage for precise focusing and displacement of the calibration plane with respect to the output fibre endface.

The system was built using the following components, as indicated in Fig. 1: DMD: ViALUX V7001 (Germany); Laser: CrystaLaser CL532-075-S (USA); ISO: Thorlabs IO-3-532-LP (USA); PMT: Thorlabs PMT2101/M; Cam: Basler piA640-210gm (Germany); BS: Thorlabs BS004; PBS: Thorlabs FPB529-23; Fibre: Thorlabs FG050LGA; Fibre patch cords: P5-488PM-FC-2; L1: AC254-060-A-ML; L2: AC254-200-A-ML; L3: AC254-075-A-ML; L5: AC254-150-A-ML; L4, L6, L9, L10: Thorlabs A240TM-A; L7: AC127-025-A-ML; L8: AC254-100-A-ML; M1-M3: Thorlabs BB1-E01; Iris: Thorlabs SM1D12D; OBJ: Olympus Plan Achromat 20X NA 0.4 (Japan); DM, Fil1, Fil2: Thorlabs filter set MDF-TOM; HW1-HW6: WPMH10M-532; QW1, QW2: WPMQ10M-532.

### Calibration methods

Binary-amplitude gratings based on the Lee hologram approach allow the DMD to be employed as a spatial light modulator in the off-axis regime^28^. The basis of input modes consists of truncated plane waves of varying *k* vectors. At the Fourier plane (focal plane of lens L2, Iris) and at the input fibre facet, this basis corresponds to a square grid of 65 × 65 focused spots. An alignment step prior to the TM acquisition measures the transmitted intensity by integrating the output speckle imaged by the CCD detector for each input mode. This step reduces the number of input modes to ~3000, since only those that are incident on the fibre core are effectively coupled. The basis of output modes consists of a square grid of 100 × 100 points spaced by ~0.5 µm across the focal plane (i.e., at a certain working distance from the output fibre facet), which are conjugate to pixels of the CCD detector during the calibration procedure. Only ~7000 output modes falling within a circular area of Ø50 µm are scanned during the image acquisition, at the maximum refresh rate of the DMD.

### Focus quality

Here, we estimate the maximum reachable power ratio *PR* – the ratio of power stored in the optimised focus and the total power leaving the output facet of the MMF. We assume that the optical system contains a set of *N* random orthogonal modes, conserves the polarisation of light and does not absorb optical power. Furthermore, we assume that we can provide a perfect phase-only modulation of the required field. The optimal field leading to the generation of a perfect focus can be expressed as an *N*-dimensional vector **f** of the complex magnitudes of the input modes in any convenient representation (e.g., focal points at the input facet or plane waves of different *k* vectors truncated at the DMD) having the real and imaginary parts $f_j = f_j^r + {\mathrm{i}}f_j^i$ obeying Gaussian statistics: $p(f^r) = \sqrt {\frac{N}{\pi }} e^{ - Nf^{r2}}$ and $p(f^i) = \sqrt {\frac{N}{\pi }} e^{ - Nf^{i2}}$. These probability distributions are normalised and scaled such that $\left| {\boldsymbol{f}} \right|^2 = \mathop {\sum }\limits_{j = 1}^N f_jf_j^ \ast = 1$. The field can also be expressed in terms of a real-positive amplitude and phase as $f_j = a_j{\mathrm{e}}^{i\varphi _j}$, where $a_j = \sqrt {f_j^{r^2} + f_j^{i^2}}$. The phase-only field is then given as $\overline {f_j} = \sqrt {\frac{1}{N}} {\mathrm{e}}^{i\varphi _j}$, where the factor $\sqrt {\frac{1}{N}}$is used so $\left| {{\bar{\boldsymbol f}}} \right|^2 = 1$, and therefore, both **f** and ${\bar{\mathbf f}}$ carry the same optical power. The power ratio *PR* is then given as the squared projection of the ${\bar{\mathbf f}}$ vector onto **f**:

1 $${PR = \left| {\mathop {\sum }\limits_{j = 1}^N \overline {f_j} f_j^ \ast } \right|^2 = \frac{1}{N}\left| {\mathop {\sum }\limits_{j = 1}^N {\mathrm{e}}^{i\varphi _j}a_j{\mathrm{e}}^{ - i\varphi _j}} \right|^2 = \frac{1}{N}\left| {\mathop {\sum }\limits_{j = 1}^N a_j} \right|^2 = N\left\langle {\boldsymbol{a}} \right\rangle ^2}$$

where **a** denotes the average value of the components of vector **a**. Given that **a** represents the radial coordinates of a 2-dimensional Gaussian distribution of complex values of vector ***f*** across the Gaussian plane, its averaged value is given by the following integral:

2 $$\left\langle {\boldsymbol{a}} \right\rangle = \mathop {\int }\limits_{ - \infty }^\infty \mathop {\int }\limits_{ - \infty }^\infty a\sqrt {\frac{N}{\pi }} e^{ - Nf^{r2}}\sqrt {\frac{N}{\pi }} e^{ - Nf^{i2}}{\mathrm{d}}f^r{\mathrm{d}}f^i \\ = 2\pi \frac{N}{\pi }\mathop {\int }\limits_0^\infty a^2e^{ - Na^2}{\mathrm{d}}a = \sqrt {\frac{\pi }{{4N}}}$$

Hence, we obtain $PR = \frac{\pi }{4}$, leading to an approximate value of 79%. Clearly, this quantity is not dependent on the number of modes supported by the optical system.

Unlike the PR, which indicates the power balance of the focus, the enhancement factor, commonly used in highly scattering random media, describes the ratio between the intensity of the optimal focus and the randomly distributed background. By considering the convenient basis of orthogonal focal points distributed across the input and output facet of the fibre, we can assume that each mode occupies the same area. Therefore, the intensity of each mode is proportional to its power. Since the fraction of total power stored in the optimised mode is given by *PR*, the combined power of all remaining (nonoptimised) modes is given as 1-*PR*, and their average intensity is $\frac{{1 - PR}}{{N - 1}} \cong \frac{{1 - PR}}{N}$. The enhancement factor $\varsigma$ is thus given by

3 $$\varsigma = \frac{{N\;PR}}{{1 - PR}}$$

Short segments of MMFs and the modulation provided by the DMD fulfil the assumptions of this evaluation very well. Although the DMD only allows for binary-amplitude modulation, when used in the off-axis regime as a Lee hologram to generate signals within an isolated zone of the Fourier space, from the perspective of *PR* and $\varsigma$, it is fully analogous to phase-only modulation, as numerous generated diffraction orders occur far from the isolated zone and do not contaminate the required optical field. Using only one circular polarisation that is well conserved during propagation, the MMF used in this study supports approximately 1050 modes, leading to a maximum enhancement factor $\varsigma$ of approximately 3800.

### Animals

Data were acquired from 5 adult mice (5-6 months old). In 4 mice, a subpopulation of inhibitory neurons, somatostatin-expressing (SST) neurons, was labelled with a red fluorescent marker (tdTomato) using a Cre-driver transgenic mouse line: Sst < tm2.1(cre)Zjh > (SST-Cre) [RRID:IMSR_JAX:013044] (Jackson Laboratory, ME, USA) crossbred with Rosa-CAG-LSL-tdTomato [RRID:IMSR_JAX:007914] mice. In 1 mouse, another subpopulation of inhibitory neurons, VIP neurons, was labelled with the same red fluorescent marker (tdTomato) using a Cre-driver transgenic mouse line: Vip < tm1(cre)Zjh > (VIP-Cre) [RRID:IMSR_JAX:010908] (Jackson Laboratory, ME, USA) cross-bred with Rosa-CAG-LSL-tdTomato [RRID:IMSR_JAX:007914] mice. The animals were housed in groups (typically 2–4 mice), and both male and female mice were used for the experiments. All procedures were approved by the University of Edinburgh Animal Welfare Committee and were performed under a UK Home Office project license.

### Surgical procedures

For craniotomy, mice were anaesthetised with isoflurane (4% for induction and 1–2% maintenance during surgery and imaging) and mounted on a stereotaxic frame (David Kopf Instruments, CA, USA). Eye cream was applied to protect the eyes (Bepanthen, Bayer, Germany), analgesics and anti-inflammatory drugs were injected subcutaneously (Vetergesic, buprenorphine, 0.1 mg/kg of body weight; carprofen, 0.15 mg; dexamethasone, 2 µg). A section of scalp was removed, and the underlying bone was cleaned before a craniotomy (approximately 2 × 2 mm) was made over the left primary visual cortex (V1, 2.5 mm lateral and 0.5 mm anterior to lambda). Cyanoacrylate glue (Locktite, UK) was applied to the surrounding skull, muscle, and wound margins to prevent further bleeding.

### In vivo imaging

The in vivo imaging stage, placed below the fibre, consisted of a custom-made frame with ear bars to hold the animal’s head position fixed during the imaging procedure and a fitted face mask for the delivery of isoflurane anaesthesia (1–2%). A suitable body temperature was maintained via a thermal bandage. The stage was mounted on a three-axis motorised translation stage with servo-driven actuators (ThorLabs, USA), allowing precise positioning of the animal both laterally for targeting the craniotomy and axially for controlling the fibre penetration process. The endoscopic fibre probe was gradually lowered into the craniotomy, up to 3 mm into the brain tissue, targeting deep cortical layers in V1 and ventrally through the hippocampus to the base of the brain. Images were collected at ~3.5 frames/s from multiple regions throughout the tissue.

At the end of the imaging session, the animals were given an overdose of sodium pentobarbital (240 mg/kg) prior to transcardial perfusion with phosphate-buffered saline (PBS) followed by 4% paraformaldehyde. The fixed brains were then extracted, and 50-µm-thick coronal sections were cut with a vibratome (Leica, Germany) to confirm the location of the fibre tract.

## Electronic supplementary material

Supplementary movie
